# Netherton syndrome - Why ENT surgeons should be aware of this rare disease - report of a case

**DOI:** 10.1186/1472-6815-13-7

**Published:** 2013-07-06

**Authors:** Kornelia EC Wirsching, Julia Heinlin, Holger G Gassner

**Affiliations:** 1 Department of Otorhinolaryngology, University Hospital Regensburg, Bavaria, Germany; 2 Department of Dermatology, University Hospital Regensburg, Bavaria, Germany

**Keywords:** Netherton syndrome, Comèl-Netherton syndrome, Facial cutaneous malignancies, Surgical facial reconstruction, Paramedian forehead flap

## Abstract

**Background:**

Comèl-Netherton syndrome is an inherited ichthyosis that is associated with highly impaired epidermal cornification and barrier function. Literature sparsely reports of the occurrence of early onset skin cancer in people with Netherton syndrome. To the best of our knowledge the suitability of the severely altered skin in patients with Netherton syndrome for techniques of facial plastic reconstructive surgery has not been discussed in literature yet.

**Case presentation:**

We present a 31-year-old caucasian female patient with Netherton syndrome who developed a defect of the right nasal ala. Biopsy revealed a well differentiated squamous cell carcinoma.

We describe the reconstruction of a full thickness nasal defect with a paramedian forehead flap and an epidermal turn-in flap in Netherton syndrome. Despite the altered skin texture, reconstruction and healing were uneventful and the surgical result was favourable.

**Conclusion:**

Therefore the authors state that the development of cutaneous malignancies should be included as a possible complication in patients with Netherton syndrome. Standard techniques of surgical facial reconstruction can be applied in these patients; healing and outcome do not appear to be negatively affected by the underlying disease.

## Background

Comèl-Netherton syndrome (= Netherton syndrome), first described in 1958, is a rare autosomal recessive disorder belonging to the group of inherited ichthyoses [1]. It is defined by three core symptoms: Congenital ichthyosiform dermatitis with defective cornification, atopic diathesis with high serum IgE levels and trichorrhexis invaginata (also called „bamboo hair“). Trichorrhexis invaginata is considered pathognomonic for the disease [2]. Chavanas et al. revealed that the genetic mutation causing Netherton syndrome is located within the SPINK5 gene on chromosome 5q32 [3]. This gene encodes the serine protease inhibitor LEKTI (for lymphoepithelial Kazal-type-related inhibitor). A lack of LEKTI weakens the skin’s protective function, thus facilitating allergen penetration and contributing to atopy in Netherton syndrome.

The manifestations of Netherton syndrome range from mild disease with ichthyosis linearis circumflexa Comèl to exfoliative erythroderma, which can end fatally within the first days postpartum. A predominance of female patients has been reported. Further complications of Netherton syndrome include mental and growth retardation and aminoaciduria. Hyperimmunoglobulinaemia E, high levels of complements (C3/C4) and a reduced number of natural killer cells make the patients susceptible for recurrent infections and allergic reactions like angioedema, hay fever and food allergies [2].

Several papers reported the development of cutaneous neoplasia in patients with inherited ichthyoses like keratitis-ichthyosis-deafness syndrome, congenital ich-thyosiform erythroderma and lamellar ichthyosis [4]. Hintner et al. were the first to describe a patient with Netherton syndrome and non-melanoma skin cancer on the back of the hand in 1980 [5]. The same patient was described by Weber et al. a couple of years later having developed multiple squamous cell carcinomas and basal cell carcinomas in the mean time [6]. Altogether we have been able to identify four patients with squamous cell carcinoma in Netherton syndrome in the literature prior to the present case [5-9]. In terms of the biomechanics of the skin-soft tissue envelope in patients with Netherton syndrome only scarce data have been published. Despite the persistent inflammatory state of the dermis, profound scarring and fibrosis is not consistently observed. No reports have been identified so far regarding the pliability and suitability of the skin-soft tissue envelope for reconstructive options in Netherton syndrome.

Standard techniques of full thickness nasal defect reconstruction include epidermal turn-in flaps and placement of paramedian forehead flap in staged procedures [10]. Each of these techniques may be severely compromised by reduced quality of the skin-soft tissue envelope. Especially the placement of a paramedian forehead flap appears fraught with uncertain success rates given the clinical presentation of the classic patient with Netherton syndrome.

In this report we present a case of nasal defect reconstruction in a young female with nasal skin malignancy in Netherton syndrome.

## Case presentation

A 31-year-old woman with Netherton syndrome diagnosed in childhood was referred to our clinic for evaluation and treatment of an atrophic defect of the right ala of the nose. The patient reported that the lesion began to develop with a small scab approximately four weeks ago and developed into an expanding, painless ulcer. She denied trauma and factitious manipulation. Patient history included previous hospital admissions for systemic and topical treatment of the underlying disease, and a consanguin marriage in former generations.

**Figure 1:**
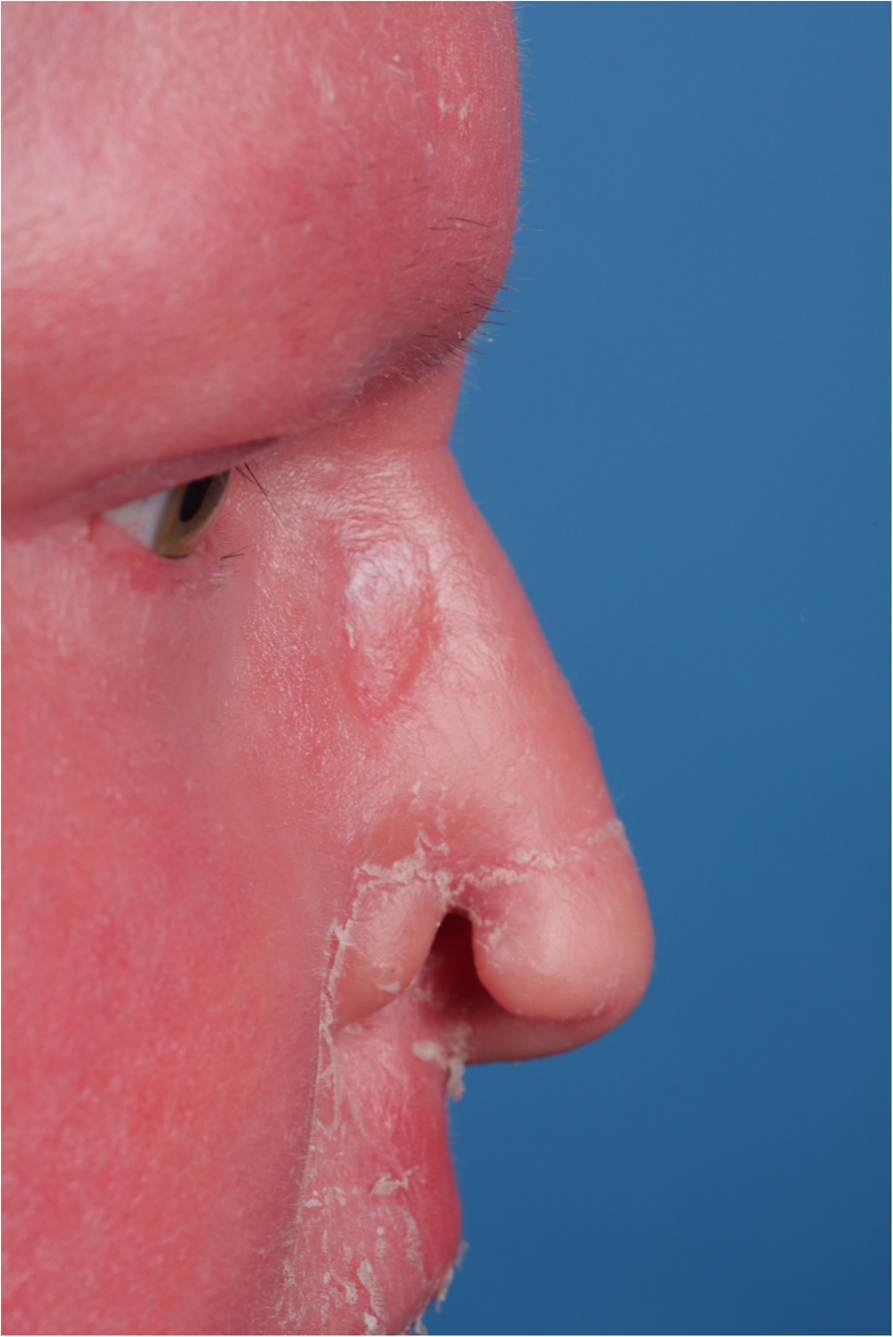
Right ala full thickness defect involving greater than 50% of the alar rim aesthetic subunit.

On clinical examination the patient’s skin was erythro-dermic, xerotic and scaling. On palpation, however, the skin-soft tissue envelope appeared soft and pliable. The patient had chapped and rough lips, easily starting to bleed when opening the mouth. Moreover she had dry, brittle and bristly hair and almost no eyebrows and lashes. There was an endophytic 4 millimeter ulcer of the right nasal ala as shown in Figure 1. Endoscopic rhinoscopy revealed the full thickness defect, but otherwise unremarkable mucosa within the nasal cavity.

The histologic evaluation of a biopsy from the edge of the defect showed a well-differentiated squamous cell carcinoma. Ultrasonography of the neck revealed no suspicious lymph nodes.

**Figure 2:**
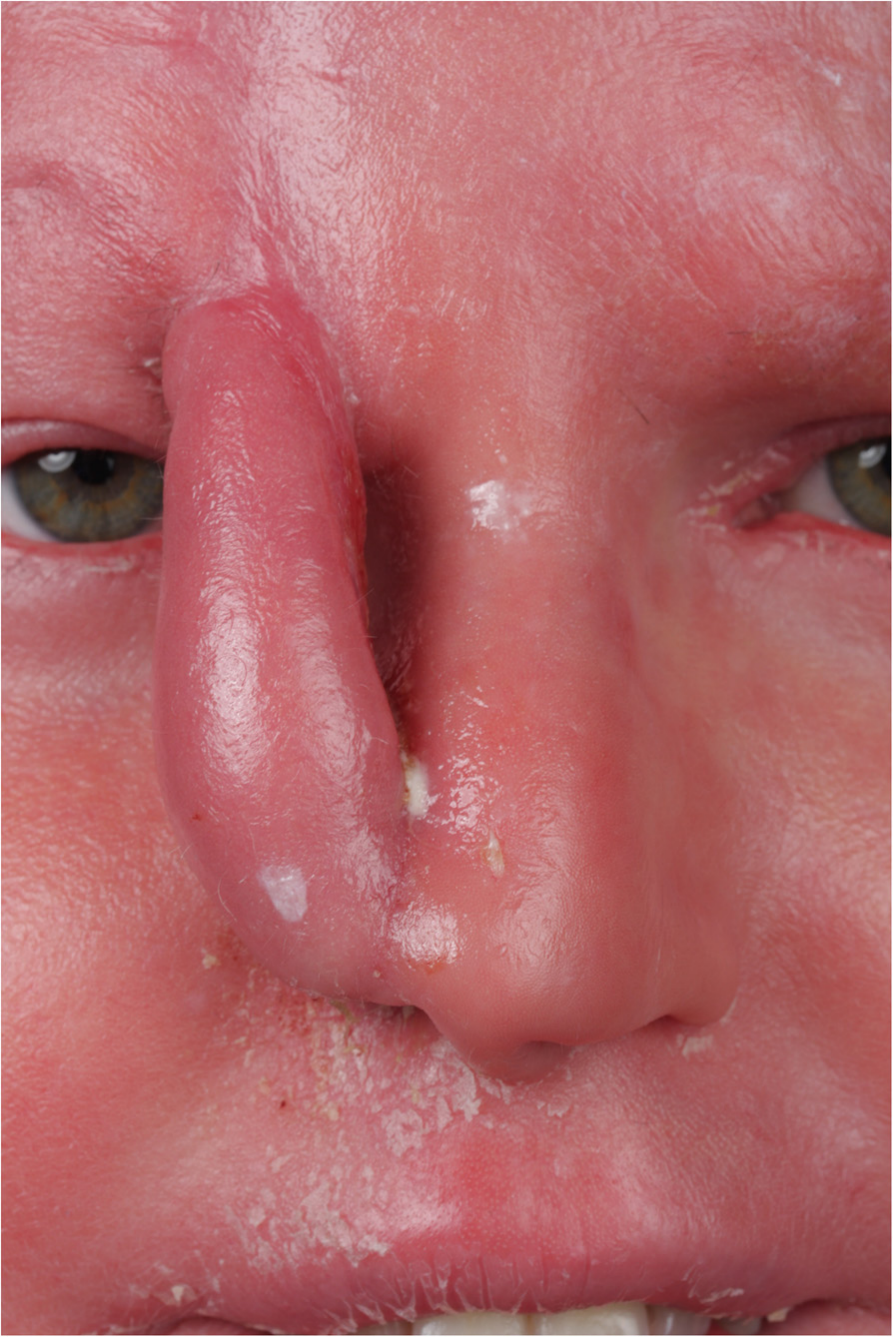
External lining reconstructed with paramedian forehead flap, three weeks after first stage procedure.

The tumor was resected under local anaesthesia, minimal margin was 4 mm on permanent histologic section. In terms of reconstruction, we discussed with the patient various options including single – stage placement of a composite graft, and a staged reconstruction with an epidermal turn-in flap and a paramedian forehead flap with the option of a third stage thinning of the flap, if needed. The patient opted for the staged approach. In the first stage, inner lining was reconstructed with two epidermal turn-in flaps of the right nasal sidewall. The skin texture and thickness appeared not ideally suited for the transposition of a nasolabial flap, and hence a paramedian forehead flap, based on the supratrochlear artery was selected for epithelial closure (Figure 2). The donor defect of the forehead was closed primarily.

**Figure 3:**
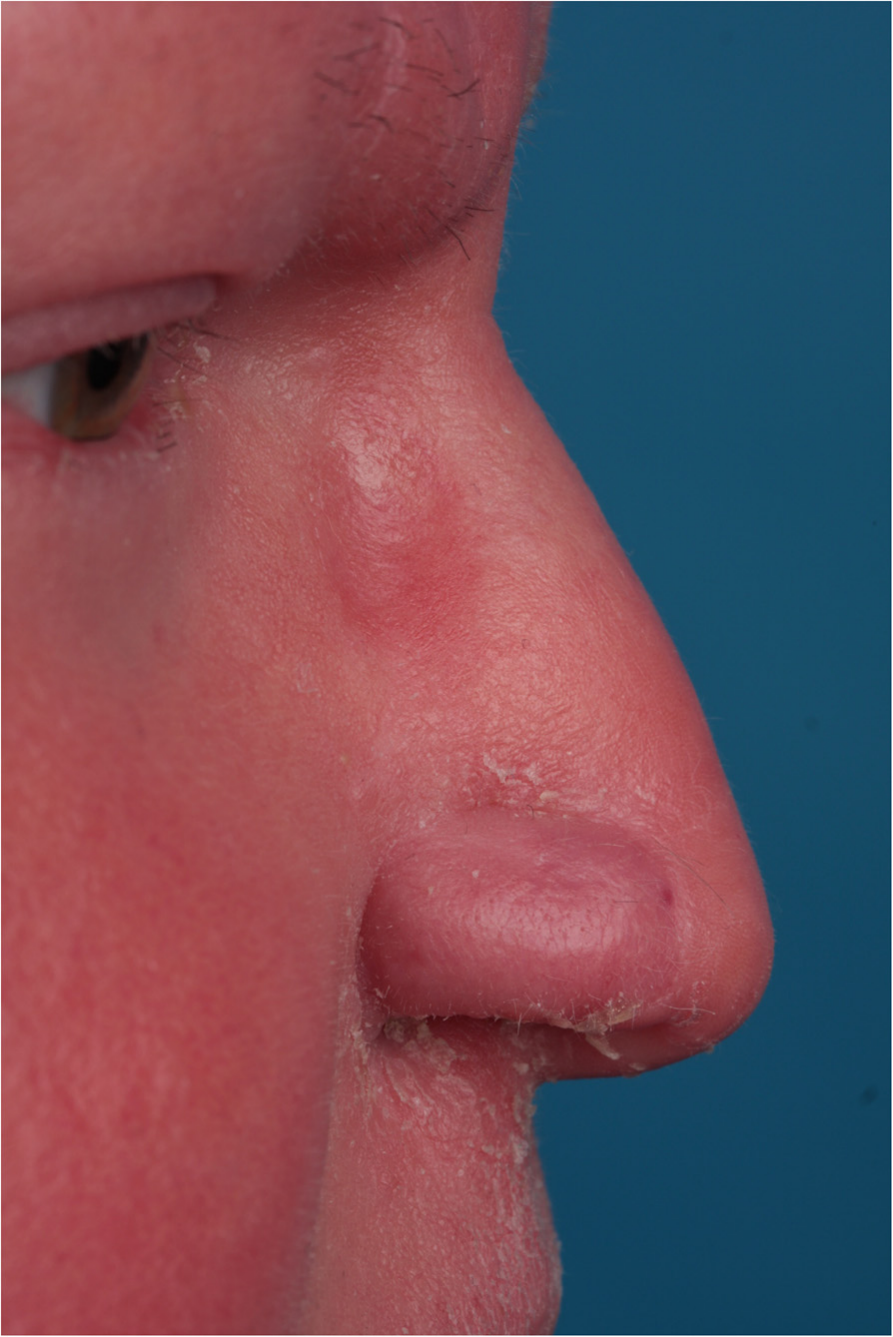
**11 months postoperative follow up.** The patient did not request a 3^rd^ stage refinement procedure to enhance the appearance of the pedicle scar.

Pedicle division and contouring of the flap was performed in the secondary constructive stage 3 weeks later under local anaesthesia. Wound healing was unremarkable. The patient has remained free of disease for 11 months follow up (Figure 3).

## Conclusions

The simultaneous presence of Netherton syndrome and epithelial cancer has been reported [5-9]. The age of the affected patients is between 23 years and 36 years, which is substantially younger than in patients without underlying disease (70 to 85years). This marked discrepancy suggests a causal relationship between the syndrome and the development of the malignancy. The definite cause of the susceptibility to squamous cell carcinoma in Netherton syndrome remains yet unknown.

Netherton syndrome may affect the T-cell differentiation leading to reduced cellular immunity. Therefore patients develop recurrent skin infections for example with Human papillomavirus (HPV). Some authors speculated about an association between the cutaneous malignancies and the presence of HPV [6]. However PCR testings in several cases failed to detect HPV DNA, showing that non-melanoma skin cancers in Netherton syndrome is not consistently caused by HPV infection. The disrupted T cell differentation furthermore impairs epidermal growth and differentiation.

Serine protease inhibitor which is affected by mutations of SPINK5 gene in Netherton syndrome, was shown to be a protective factor in breast cancer. A lack of serine protease inhibitor impairs the skins potential of DNA repair and therefore possibly also contributes to cutaneous carcinogenesis [7].

Another factor for the susceptibility to squamous cell carcinoma in Netherton syndrome patients could be the multiple treatments with aggressive medication like in-terferon-alpha, retinoids, methotrexate, corticosteroids or PUVA (psoralen plus ultraviolet A (UVA)), which is known to induce mutations of the tumor suppressor protein p53 [8]. All these factors together may have contributed to the unusual occurrence of squamous cell carcinoma on the nose of our patient with this rare skin disorder.

Frequent infections and the extensive use of topical and systemic medication may adversely affect wound healing. In addition, the chronic state of inflammation suggests a less than optimal environment for facial reconstructive surgery. On the other hand, the palpable softness and pliability of the skin-soft tissue envelope in the present case encouraged the consideration of surgical reconstructive options. Neither technique of dissection nor timing of surgical staging or post-operative wound care were altered in the present case. Wound healing was unremarkable in the sense that the chronic erythema and scaling of the skin remained stable and no delays or deficits of healing were observed. The cosmetic and functional results appeared very favorable (Figure 3).

A review of literature as well as the present case suggests an increased awareness for the development of skin cancers in Netherton syndrome. The approach to facial reconstruction in these patients seems not to require fundamental modification despite the clinically obvious marked inflammation of the skin with substantial erythema and scaling. Favorable functional and cosmetic results may be obtained in these patients.

### Consent

Written consent, for publication of the clinical details and clinical images was obtained from the patient. A copy of the consent form is available for review by the Editor of this journal.

## Competing interests

The authors declare that they have no competing interests.

## Authors’ contributions

KW carried out the writing of the manuscript, acquisition of informed consent and literature research and approval of all images in the case report. JH helped with her dermatologic background knowledge in outlining and modification of the manuscript. HG supervised, commented and helped in the revision of the manuscript as well as the selection of photographs used in the case report. All authors have read and approved the final manuscript.

## Pre-publication history

The pre-publication history for this paper can be accessed here:

http://www.biomedcentral.com/1472-6815/13/7/prepub
